# *Agrobacterium* Uses a Unique Ligand-Binding Mode for Trapping Opines and Acquiring A Competitive Advantage in the Niche Construction on Plant Host

**DOI:** 10.1371/journal.ppat.1004444

**Published:** 2014-10-09

**Authors:** Julien Lang, Armelle Vigouroux, Sara Planamente, Abbas El Sahili, Pauline Blin, Magali Aumont-Nicaise, Yves Dessaux, Solange Moréra, Denis Faure

**Affiliations:** 1 Institut des Sciences du Végétal (ISV), UPR2355, CNRS, Saclay Plant sciences, Gif-sur-Yvette, France; 2 Laboratoire d'Enzymologie et Biochimie Structurales (LEBS) UPR3082, CNRS, Gif-sur-Yvette, France; 3 Institut de Biochimie et de Biophysique Moléculaire et Cellulaire, UMR8619, CNRS, Université Paris-Sud, Orsay, France; University of Toronto, Canada

## Abstract

By modifying the nuclear genome of its host, the plant pathogen *Agrobacterium tumefaciens* induces the development of plant tumours in which it proliferates. The transformed plant tissues accumulate uncommon low molecular weight compounds called opines that are growth substrates for *A. tumefaciens*. In the pathogen-induced niche (the plant tumour), a selective advantage conferred by opine assimilation has been hypothesized, but not experimentally demonstrated. Here, using genetics and structural biology, we deciphered how the pathogen is able to bind opines and use them to efficiently compete in the plant tumour. We report high resolution X-ray structures of the periplasmic binding protein (PBP) NocT unliganded and liganded with the opine nopaline (a condensation product of arginine and α-ketoglurate) and its lactam derivative pyronopaline. NocT exhibited an affinity for pyronopaline (*K_D_* of 0.6 µM) greater than that for nopaline (*K_D_* of 3.7 µM). Although the binding-mode of the arginine part of nopaline/pyronopaline in NocT resembled that of arginine in other PBPs, affinity measurement by two different techniques showed that NocT did not bind arginine. In contrast, NocT presented specific residues such as M117 to stabilize the bound opines. NocT relatives that exhibit the nopaline/pyronopaline-binding mode were only found in genomes of the genus *Agrobacterium*. Transcriptomics and reverse genetics revealed that *A. tumefaciens* uses the same pathway for assimilating nopaline and pyronopaline. Fitness measurements showed that NocT is required for a competitive colonization of the plant tumour by *A. tumefaciens*. Moreover, even though the Ti-plasmid conjugal transfer was not regulated by nopaline, the competitive advantage gained by the nopaline-assimilating Ti-plasmid donors led to a preferential horizontal propagation of this Ti-plasmid amongst the agrobacteria colonizing the plant-tumour niche. This work provided structural and genetic evidences to support the niche construction paradigm in bacterial pathogens.

## Author Summary

An ecological niche is defined, in a given environment, by the availability of nutritive resources, which can be specifically assimilated by certain living organisms to promote their proliferation. The bacterial pathogen *Agrobacterium tumefaciens* is able to engineer an ecological niche in the infected host via the transformation of the plant genome and diversion of the plant metabolism towards production of the opine nutrients. In this work, we quantified the selective advantage conferred to a member of the phytopathogenic species *A. tumefaciens* which is able to assimilate the opine nopaline. This opine is a condensate of arginine and α-ketoglurate that is produced both under linear and cyclic forms in the plant tumour environment. We further determined at the molecular and atomistic levels how *A. tumefaciens* is able to sense the nopaline molecules, and which metabolic pathways are activated in response. Overall, this work deciphered some key molecular events in the niche construction of the pathogen *A. tumefaciens* that is unique among living organisms and used to develop bioengineering tools.

## Introduction

The widespread pathogen *Agrobacterium tumefaciens* evolved a unique process of niche construction inside the host plant: it transfers a portion (T-DNA) of its tumour-inducing (Ti) plasmid into the nuclear genome of the infected plant cells [1], [2]. In the stably transformed host cells, the expression of the T-DNA genes drives synthesis of the plant growth factors auxin and cytokinins that enhance cell proliferation and vascular differentiation. In parallel, the surface and intercellular spaces of the neoplastic tissues of the tumour - that is a characteristic symptom of the crow-gall disease - are colonized by the bacterial pathogen. The carbon and nitrogen metabolisms of the transformed cells are also diverted. The T-DNA codes for the enzymatic synthesis of unusual metabolites. These diverse derivatives of sugars, phosphorylated or not, or amino, or organic acids are called opines [3]–[6]. As an example, the well-studied *A. tumefaciens* strain C58 induces accumulation of different opines such as nopaline that is formed from arginine and α-ketoglutarate (α-KG) and agrocinopines A and B that are non-nitrogenous phosphodiesters of sugars.

Besides the opine synthesis genes carried on the T-DNA, the Ti-plasmid also harbours genes involved in the uptake and catabolism of the opines by *A. tumefaciens*. In other words, the bacteria carrying a Ti-plasmid can benefit from the opines produced by the cells of the plant tumours that express the appropriate T-DNA genes. Aside, some opines, the so-called conjugative opines such as agrocinopine, trigger the quorum-sensing pathway which in turns activates the horizontal transfer (bacterial conjugation) of the Ti-plasmid [7]. As a consequence, the tumour niche was called the opine niche, and the niche construction process was theorized as the opine concept [3], [4]. This concept highlights the key-role of opines in the maintenance and dissemination of the Ti-plasmids among the agrobacteria colonizing the plant host. Experimental evaluation of the validity of the opine concept was performed by measuring the selective advantage conferred to opine-assimilating bacteria, including agrobacteria and other species, in opine-rich environments, such as a culture medium or the rhizosphere and phyllosphere of opine-producing plants [8]–[13]. However, to our knowledge, this hypothesis has not been tested in the natural opine niche, i.e. the plant tumour, making the opine concept in the *Agrobacterium*-plant host interaction formally still unproven.

In *A. tumefaciens*, the recognition and import of opines is conferred by periplasmic binding proteins (PBPs) and their associated ATP-binding cassette (ABC) transporters [14], [15]; but there is no known structure of the opine-PBP complex. In this work, we investigated the structural and biochemical properties of the PBP NocT of *A. tumefaciens* C58 which senses at least two opines: nopaline and pyronopaline, a lactam nopaline-derivative that can also be found in plant tumours [16], [17]. In addition, the complete set of *A. tumefaciens* genes transcriptionally responsive to these opines was identified and the fitness advantage conferred by opine assimilation upon *A. tumefaciens* was measured directly in the plant tumours. This integrative work highlights the structural and functional characteristics of opine binding and assimilation in the niche construction process evolved by the plant pathogen. It also definitely provides evidence to support the opine concept that directs the *A. tumefaciens* plant-host interaction.

## Results

### Structures and overall fold of NocT

**Figure 1:**
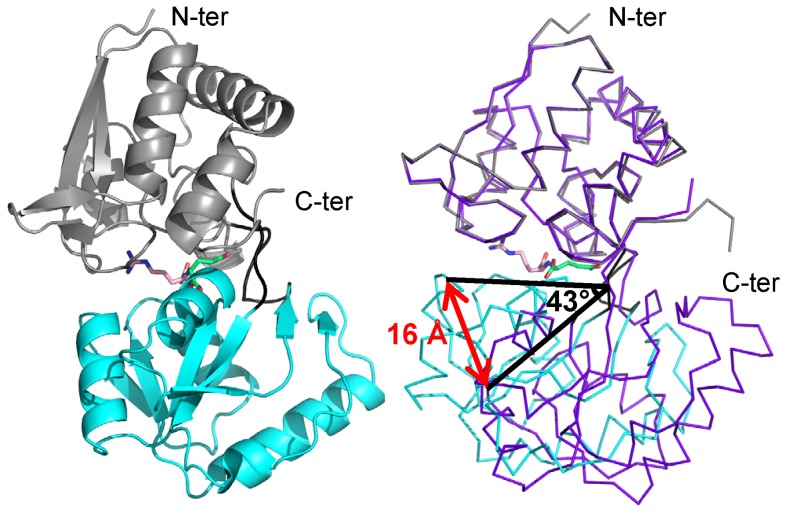
Ribbon representation of NocT in complex with nopaline and comparison with the unliganded form. Left panel; Nopaline is located in the cleft between the two domains shown in grey and cyan. The short hinge region between the two domains is shown in black. The arginine part of the nopaline is shown in pink while its α-KG part in limegreen. Right panel, comparison of the unliganded structure of NocT in purple with the complexed nopaline in grey and cyan.

The X-ray structures of the mature free-liganded NocT and the two liganded NocT with pyronopaline and nopaline were obtained at 1.89 Å, 1.55 Å and 2.29 Å resolutions, respectively (Table S1). Each crystal contained two very similar molecules in the asymmetric unit as indicated by the overall root mean square deviations (RMSD) for all Cα atoms of 0.6 Å, 0.46 Å, and 0.28 Å respectively. The two liganded forms adopted a similar closed conformation (RMSD of 0.3 Å for all Cα) while the unliganded form showed an open conformation (Figure 1) as commonly reported for the structures of PBPs solved with and without a ligand [18]–[20]. A 43° rotation around the hinge region of the C-terminal domain (residues 117–231) was observed once the N-terminal domains (residues 29–112 and 239–284) of the unliganded and liganded structures were superimposed leading to a movement of 16 Å for Thr168. NocT possesses a typical fold of cluster F within the PBP structural classification [21]. Indeed, a structural comparison of its closed form to all entries in the PDB using SSM-EBI (http://www.ebi.ac.uk/msd-srv/ssm [22]) showed that the most similar overall structures were PBPs from the same cluster F, i.e. the liganded structure of the histidine binding protein HisJ (PDB code 1HSL) of *Escherichia coli* and that of the lysine-arginine-ornithine binding protein LAO (PDB code 1LAF) of *Salmonella enterica* [23], [24]. RMSD values of NocT vs. HisJ and LAO ranged from 1.47 to 1.5 Å over 226/228 Cα atoms, corresponding to a sequence identity of 38 and 36% respectively.

### NocT ligand binding site with nopaline and pyronopaline

**Figure 2:**
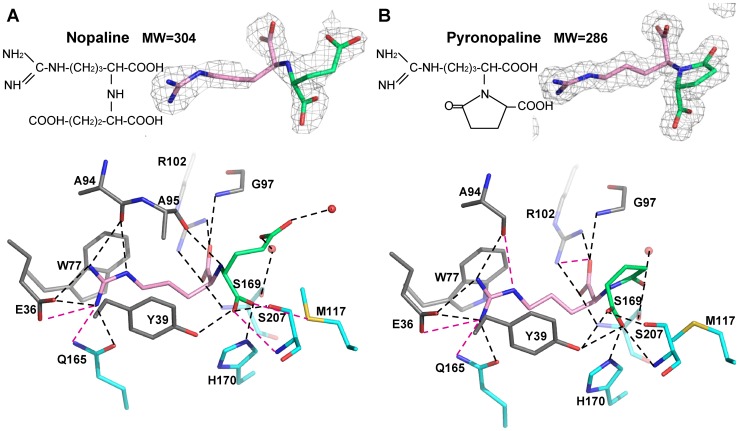
Nopaline (A) and pyronopaline (B) bound to the ligand binding site of NocT are shown as pink/limegreen and pink/blue stick in their simulated annealing Fo-Fc omit map contoured at 4 σ, respectively. Hydrogen bonds between NocT and the ligand are shown as dashed lines in black (for distances below 3.2 Å) and magenta (for distances between 3.2 and 3.4 Å).

The nopaline and pyronopaline bound between the two closed lobes were very well defined in their respective electron density maps (Figure 2). The ligand-binding site of NocT was defined by the Glu36, Tyr39, Tyr42, Trp77, Ala94, Ala95, Gly97, Arg102, Thr115, Met117, Gln165, Thr168, Ser169, His170, Ser207, Gly238 and Val239 residues. Overall both ligands shared the same protein binding mode for their arginine moiety. The side chain of this moiety was wedged between two aromatic residues (Trp77 and Tyr39) and pointed toward the opening of the cleft by making seven hydrogen bonds with the side chains of Gln165 and Glu36 and the carbonyl of Ala94 (Figure 2). Its carboxyl group made a salt-bridge with the side chain of Arg102 and interacted with both NH of Gly97 and Ser169 while its NH group formed a hydrogen bond with the carbonyl of Ala95. Remarkably this hydrogen bond cannot exist with pyronopaline due to the lactam structure of the molecule.

Concerning the α-KG parts of both ligands, their first carboxyl group adopted a different position (shift around 1 Å of the two oxygen atoms), but made similar hydrogen bonds with NocT involving the side chains of Tyr39, His170 and Ser207 as well as the NH of Ser207. An additional bond was observed between the Met117 side chain and the nopaline. In the pyronopaline, the hydroxyl of the terminal carbon covalently linked to the N atom interacted with the side chain of Ser169 whereas in the nopaline, each oxygen from the terminal carboxylate group of the α-KG moiety formed two water-mediated interactions with the main chains of Thr115 for one water and with the CO of Phe235 and the S169 side chain for the other water (Figure S1). An analogous water molecule interacting with the CO main chain of Phe235 in the pyronopaline-liganded structure was shifted by 1.4 Å and bound the Gln99 side chain which appeared well ordered in this structure. The large mobile residue Met117 was forced to accommodate its side chain position according to the type of bound ligand and made Van der Walls contacts with the pyronopaline (Figure S1). The rearrangement of the side chain (shift of 1.9 Å) in the pyronopaline structure compared with the nopaline structure led to a rearrangement of the carboxyl moiety of the α-KG and of the H170 side chain (Figure S1).

### Affinity of NocT to nopaline and pyronopaline

By intrinsic protein fluorescence titration, the dissociation constant (*K_D_*) values between NocT and nopaline and pyronopaline were 3.7±0.6 and 0.57±0.07 µM respectively (Figure S2). These apparent *K_D_* values were in the low micromolar range usually observed for PBP ligands [21]. Using isothermal titration calorimetry (ITC), the mean *K_D_* value between NocT and pyronopaline was determined as 0.58±0.05 µM (Figure S2), identical to that obtained by fluorescence measurement. The ITC data also confirmed the 1∶1 binding stoichiometry and demonstrated a positive enthalpy change upon pyronopaline binding. This binding reaction was entirely driven by a large favorable increase in entropy (TΔS = 12.6 kcal/mol). With both fluorescence titration and ITC techniques, no interaction could be measured between NocT and arginine; ITC also revealed no interaction between NocT and histidine or ornithine (Figure S2).

### Comparison of the ligand binding site of NocT with other PBPs and nopaline-binding signature

**Figure 3:**
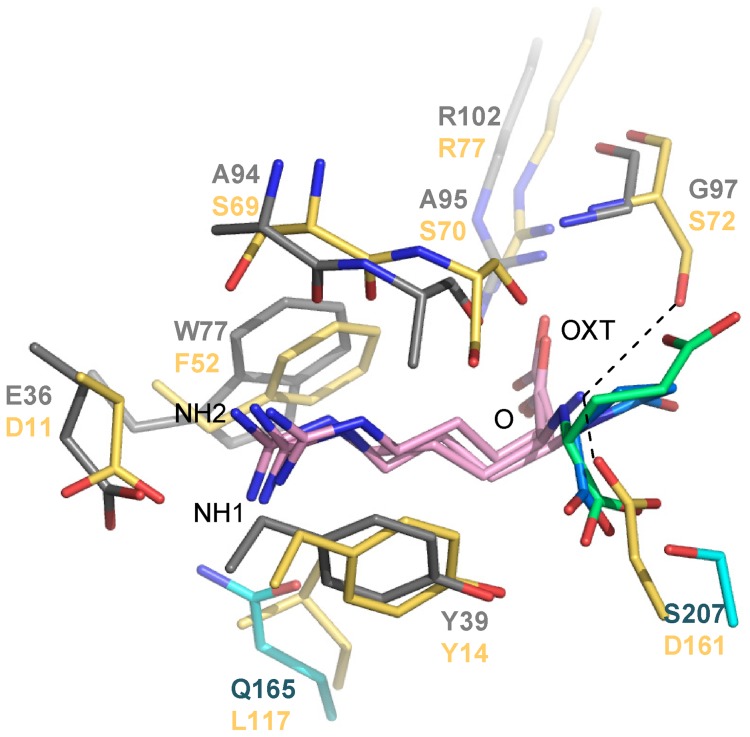
Structural comparison between the binding sites of NocT (shown in color domains as in **Figure 1**) in complex with nopaline and pyronopaline and the *Salmonella* PBP LAO in complex with arginine (PDB code 1LAF; in yellow). Nopaline, pyronopaline and arginine ligands are shown in pink/limegreen, pink/blue and pink respectively.

Around the arginine moiety of the nopaline/pyronopaline ligand, the ligand binding site of NocT resembled those of the PBPs HisJ and LAO which have been shown to bind arginine and other amino acids (Figure 3). Two residues were conserved in NocT, HisJ and LAO: Tyr39 (Tyr14 in LAO) which stacked the arginine side chain ligand and Arg102 (Arg77 in LAO) which bound the carboxyl group of the arginine ligand. Glu36 was replaced by an equivalent Asp residue that preserved the interaction with the guanidinium group of the ligand. The position of the side chain arginine ligand and that of its carboxyl group were similar in the three PBPs leading to similar polar interactions. The amino group of the arginine ligand was tightly bound in HisJ and LOA with the side chains of a serine at position 72 (corresponding to Gly97 in NocT) and an aspartate at position 161 (Ser207 in NocT). Importantly, these two major interactions will be lost in NocT leading to a less efficient binding of an arginine ligand in line with the observed absence of detectable interaction between NocT and arginine by ITC and fluorescence titration. The presence of Gly97 in NocT seems especially essential for the spatial accommodation of the α-KG moiety of nopaline/pyronopaline whereas the equivalent residue (Ser72) in HisJ and LAO seems incompatible with nopaline or pyronopaline binding due to steric clash.

In *A. tumefaciens* strains harbouring an octopine-type Ti plasmid, the PBP OccJ allows the uptake of the opine octopine which is a condensate of arginine and pyruvate [25]. Interestingly, NocT seems involved in the importation of nopaline and octopine (only in the presence of nopaline), whereas OccJ permits the importation of octopine but not that of nopaline ([25], [26]. The structure of OccJ is unknown. Sequence comparison between NocT and OccJ shows that among the 12 residues of NocT which directly interact with nopaline/pyronopaline, 4 are different in OccJ. These are G97, M117, H170 and S207 in NocT which correspond to serine, asparagine, alanine and asparagine residues in OccJ, respectively. As mentioned above in the NocT/LAO/HisJ comparison, the replacement of Gly97 in NocT by a serine at the equivalent position might be responsible for preventing OccJ from binding nopaline. Moreover M117, H170 and S207 interact only with the α-KG moiety of nopaline/pyronopaline. We therefore define these 4 amino acids G_97_M_117_H_170_S_207_ as the nopaline-binding signature.

To learn more on the role of Met117 residue in the interaction between NocT and its ligand, we constructed two NocT mutants with different short polar residues: NocT-M117N because an asparagine is found in the OccJ sequence and NocT-M117S because a serine is found in the LAO/HisJ sequences. Both mutants displayed a similar *K_D_* for pyronopaline that was 76-fold higher than that of the WT protein, proving that Met117 is a key component of the ligand affinity and belongs to the nopaline-binding signature. We also obtained the structure of NocT-M117N in complex with pyronopaline (Table S1). Prior to this, the stability of NocT-M117N was verified using differential scanning calorimetry (DSC) and, compared to the WT protein, the mutant presented similar Tm (60.12°C versus 60.25°C for the WT) and ΔH (1.59 10^5^ cal/mol/°C versus 1.4 10^5^ cal/mol/°C for the WT) (Figure S3), meaning that mutating M117 has no significant effect on protein stability. The structure of NocT-M117N in complex with pyronopaline confirmed that the replacement of Met117 with Asn led to a loss of hydrophobic interaction with the ligand and showed that His170 which is free to move towards Asn117 bound the carboxyl group of the α-KG of the pyronopaline in a position not observed in the WT complexes (Figure S4). A loss of the polar interaction between the side chain of Ser169 and the terminal OH of the pyronopaline ring was also observed due to steric hindrance.

### The NocT phylogenetical cluster is branched to amino acid-binding PBPs

**Figure 4:**
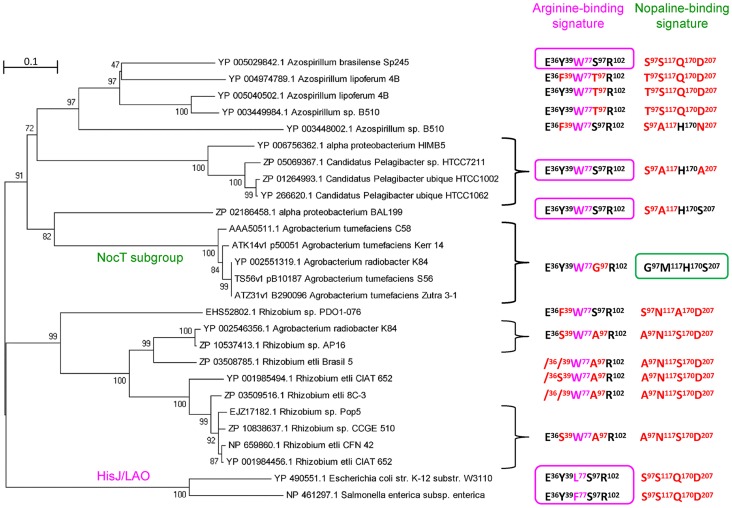
NocT phylogeny and occurence of the nopaline-binding signature. The displayed subtree was rooted with HisJ and LAO sequences. For each protein or protein cluster, the residues which are identical to (black) and different from (red) those involved in the binding of arginine (pink box) and nopaline (green box) are indicated. The W, F and L residues at position 77 (pink) may play a similar role in maintaining arginine.

Five hundred bacterial NocT-homologous PBPs with a threshold set at at least 40% of identity were recovered using blastp at NCBI. To these were added 53 other homologous sequences found in the *Agrobacterium* genomes of the AgrobacterScope genome library (Genoscope, France). The relation tree constructed from these 553 sequences revealed different subgroups. The closest NocT relatives were used to build a novel relation tree rooted with the HisJ and LAO sequences (Figure 4). Members of the NocT subgroup were highly similar PBPs which belong to the *A. tumefaciens* strains C58, S56, Zutra3-1, Kerr14 (biovar 1), and *A. radiobacter* K84 (biovar 2). All of them are nopaline-assimilating agrobacteria. Their genomes exhibited a strong synteny with respect of the *noc* operon region of *A. tumefaciens* C58. Moreover, all these NocT proteins retained the nopaline-binding signature G_97_M_117_H_170_S_207_. Outside the NocT subgroup, the nopaline-binding signature was strongly degenerated, but the arginine-binding signature was conserved in some proteins, such as one PBP of the marine alpha-proteobacterium BAL199 (NCBI BioProject PRJNA54661). These features suggested that the ability to bind nopaline was highly specific to members of the NocT PBP-subgroup, and that NocT and some arginine-PBPs could have evolved from a common ancestor.

### Nopaline/pyronopaline regulon in *A. tumefaciens* C58

**Figure 5:**
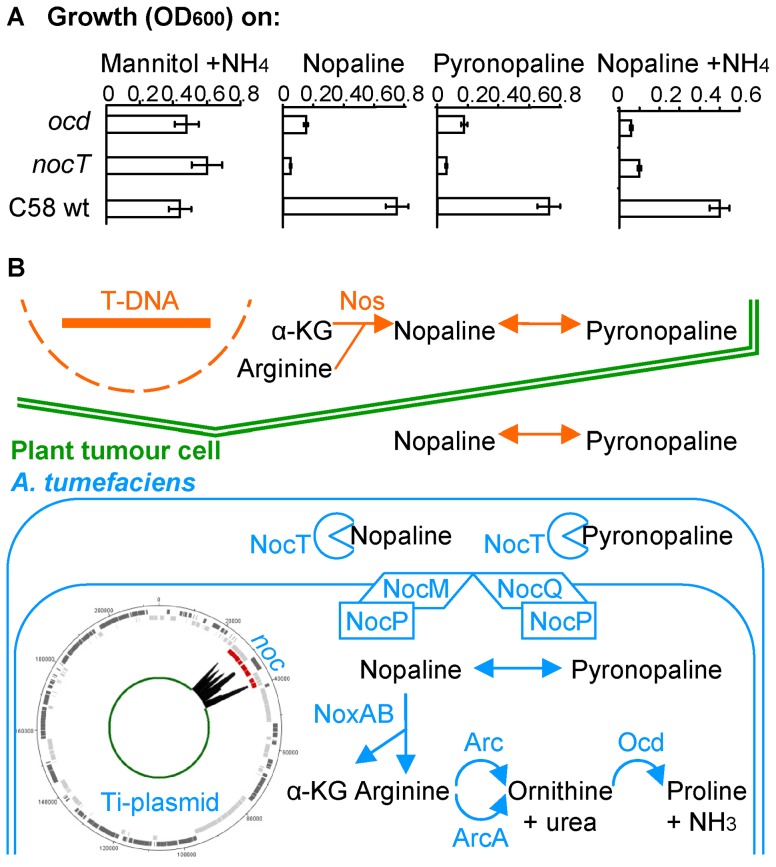
The nopaline/pyronopaline regulon in *A. tumefaciens* C58. A, growth of the *A. tumefaciens* C58 wt and *nocT* and *ocd* mutants on AB medium supplemented with mannitol and NH_4_, or nopaline or pyronopaline as sole sources of carbon and nitrogen. The OD_600_ values were taken after 24 hours; B, schematic representation of the nopaline/pyronopaline synthesis and degradation pathways in the plant tumour. The NocPTQM (ABC transporter), NoxAB (nopaline oxidase A and B), Arc (arginase) and Ocd (ornithine cyclodeaminase) proteins are encoded in the *noc* region of the Ti-plasmid. A transcriptome graph indicates the position and expression fold change of the *noc* genes on the Ti-plasmid in presence of nopaline/pyronopaline. The ArcA protein is encoded in the linear chromosome. Spontaneous conversion between nopaline and pyronopaline is represented by double arrows.

The capability of NocT to bind pyronopaline suggested that *A. tumefaciens* C58 could use the same pathway for the assimilation of nopaline and pyronopaline. To test this hypothesis, we constructed two *A. tumefaciens* C58 KO-mutants that were affected in the binding and transport (*nocT*) and catabolism (*ocd*) of nopaline. When nopaline or pyronopaline were used as sole sources of carbon and nitrogen and when nopaline was used as a sole source of carbon in the presence of inorganic nitrogen, the *nocT* mutant could not grow while the growth of *ocd* mutant was strongly impaired (Figure 5A). These features established that nopaline and pyronopaline were assimilated by the same catabolic pathway Noc, which had been previously characterized for nopaline [27]. In this pathway (Figure 5B), the nopaline is cleaved into α-KG and arginine by the enzymatic complex NoxAB; then, the degradation of arginine into proline is completed by the enzymes Arc, ArcA and Ocd, and the conversion of proline into glutamate, a C and N-source, by the enzyme PutA ( = Atu4157) [27]–[30].

Furthermore, we delineated the nopaline and pyronopaline regulon by transcriptomics. In this experiment, an *accR* mutant of *A. tumefaciens* C58 was used because this mutant expresses the transcriptional regulator NocR at a higher level than does the wild type strain [31]. In presence of nopaline NocR induces the expression of the *noc* genes [32]. Consequently, an optimal expression of the nopaline regulon was expected in the *accR* genetic background. Noticeably, the *accR* mutant also mimics the condition of an exposure to the opine agrocinopine that accumulates in *A. tumefaciens* C58-induced plant tumours [33]. Comparative transcriptomics in cell culture of an *accR* mutant grown in the presence or absence of nopaline and pyronopaline should therefore reveal nopaline/pyronoplaine responsive genes in a context reminiscent of the plant tumour environment. With this approach, 32 differentially (Fch>3; P<0.05) expressed genes of *A. tumefaciens* C58 were identified in the presence of nopaline/pyronopaline, 8 of them being downregulated and 24 upregulated (Table S2). Subsequent RT-qPCR experiments on a selection of genes (*cysJ*, *arcA*, *nocT*, *nocP* and *noxB*) confirmed the micro-array results (Table S2). The highest upregulated genes (Fch>20) included the *noc* operons of the Ti-plasmid [14] and an arginase-encoding gene (*arcA* = *atu4007*) located on the linear chromosome. As no other catabolic functions were affected, we suggest to refer to these highly-expressed genes as the nopaline/pyronopaline core-regulon (Table S2).

### NocT-mediated selective advantages in the plant-tumour niche

**Figure 6:**
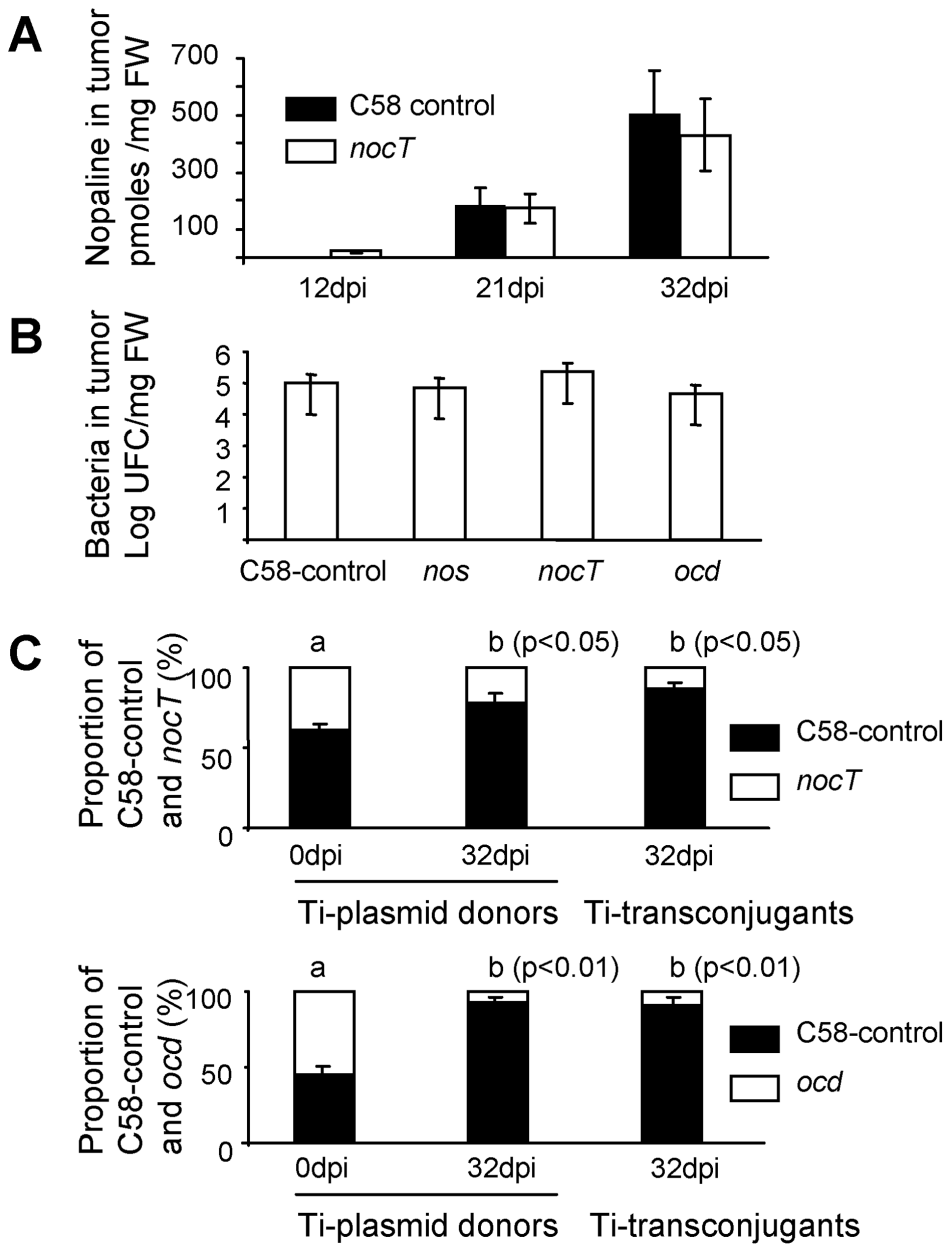
Involvement of NocT in the fitness of *A. tumefaciens* in the plant tumour. A, nopaline level in plant tumours induced by *A. tumefaciens* C58-control and *nocT* derivative; B, cell number of *A. tumefaciens* C58-control and its *nos*, *nocT* and *ocd* derivatives when they individually colonize plant tumour; C, competitive infection between the *A. tumefaciens* C58-control strain and *nocT* or *ocd* derivatives, all acting as Ti-plasmid donors in the presence of the recipient *A. tumefaciens* C58.00. Proportion (%) of the Ti-plasmid genotypes among donor strains in the inoculum (0 dpi) and the mature tumours (32 dpi) and among transconjugants (32 dpi) is indicated. Exact Fischer test was used for statistical analysis (p-value thresholds are indicated on the graph).

In the tumours induced on tomato plants, we measured the level of nopaline and pyronopaline. Nopaline but not pyronopaline could be detected by mass spectrometry. Moreover, nopaline accumulated at significant and equivalent levels in tumour tissues induced by the C58-control and by the *nocT* strains (Figure 6A). We therefore confirmed that the tumour niche is a natural nopaline-rich environment. However, as the quantification of nopaline was performed on whole plant tumour extracts, it remains possible that only a small fraction of this nopaline is available to the *A. tumefaciens* cells colonizing the exterior surface of the tumour.

Thereafter, we investigated the selective advantage conferred upon *A. tumefaciens* C58 by the nopaline pathway in plant tumours. In addition to the *nocT* and *ocd* mutants that were impaired in the assimilation of nopaline, we constructed a *nos* mutant that was defective for the synthesis of the nopaline by the tumour cells (see nopaline pathway in the Figure 5). When these mutants were individually used to inoculate tomato plants, they colonized the tumour tissues at population sizes (10^5^ CFU/mg FW) similar to that reached by the C58-control strain (Figure 6B). Hence, the tumour environment provides enough nutrients to support the growth of the pathogens whatever their capacities to produce, transport or catabolize the nopaline and pyronopaline.

In a second experiment, the *nos*, *nocT* and *ocd* KO-mutants were co-inoculated with the C58-control strain in presence of the recipient strain C58.00 (free of Ti and At plasmids). For each of these conditions the mixed populations colonized the plant tumours at levels (total cell number at 10^5^ CFU/mg FW) that were similar to those reached in the single infections. The frequency of Ti-plasmid donors was determined at the infection time and in the plant tumours to allow the calculation of competitive indexes (CI, see materials and methods). As expected, the CI value reached 1.0 in the competition between the *nos* mutant and C58-control. In this case, nopaline that is synthesized via the expression of the wild-type T-DNA gene, benefited equally to the two co-inoculated nopaline-utilizing populations (*nos* mutant and C58-control). In contrast, CI values were only 0.35 and 0.07 in the competitions that involved *nocT* or *ocd* mutants and C58-control, respectively. These low CI values revealed that the C58-control population significantly outcompeted *nocT* and *ocd* populations which were unable to assimilate nopaline (Figure 6C).

Unlike agrocinopines A and B, nopaline is not a conjugative opine in *A. tumefaciens* C58, hence the regulation of the plasmid Ti-transfer genes is independent of the presence and concentration of nopaline. In the above plant tumour assays, we were therefore also able to evaluate the capacity of each of the competitors to disseminate its Ti-plasmid into the recipient strain C58.00. In plant tumours, Ti-plasmid transconjugants reached up to 10^2^ CFU/mg FW. The proportion of transconjugants which acquired the Ti-plasmid from each of the two donors in competition was measured (Figure 6C). We observed that, among transconjugants, the relative abundance of the Ti-plasmid that conferred nopaline-assimilation was higher than that of the Ti-plasmid that did not confer nopaline-assimilation. Preferential accumulation of the nopaline-using transconjugants we observed could simply mirror the relative abundance of Ti-plasmid donors in the tumour niche. The competitive advantage which is conferred by opine assimilation could therefore also contribute to dominance of opine-using transconjugants over non-users.

## Discussion

This work revealed the structural basis of the PBP NocT that is required for the binding and assimilation of the opines nopaline and pyronopaline in *A. tumefaciens* C58. Structural and amino-acid sequence analyses showed that NocT was unique among more than 150 PBPs that are encoded by the *A. tumefaciens* C58 genome [34]. Genome data-base analysis also showed that close homologues of NocT can be only found in the nopaline-assimilating strains of the genus *Agrobacterium*: either in the pathogenic strains C58, S56, Zutra 3-1, and Kerr 14 belonging to the *A. tumefaciens* species complex, or in non-pathogenic ones such as the biocontrol strain *A. radiobacter* K84 which carries the nopaline binding *nocT* gene on a 185-kbp plasmid [35]. In combination with toxin production [36], [37], nopaline assimilation might contribute to the capacity of *A. radiobacter* K84 to compete with *A. tumefaciens* pathogens in the plant environment. Based on our data we cannot rule out the possibility that other nopaline-assimilating isolates of different *Agrobacterium* species for whom genome sequence is still unknown could also possess a NocT PBP [38], [39].

Previous reports established that the two PBPs LAO (*Salmonella*) and HisJ (*Escherichia*), structurally close to NocT, can bind basic amino acids such as arginine [23], [24]. The resemblance between NocT and LAO/HisJ structures and ligand binding sites is not surprising because nopaline and pyronopaline are L-arginine derivatives. However, we showed here that NocT cannot bind arginine (nor histidine and ornithine) probably due to the absence of two essential side chains (Ser72 and Asp161 in LAO corresponding to Gly97 and Ser207 in NocT), both locking the α-amine. The absence of binding between arginine and NocT might confer two advantages. First it might prevent arginine from competing with nopaline for uptake by the Noc system and second it might prevent NocT from becoming saturated with arginine if it cannot pass on to the nopaline transporter. In parallel the presence of a Ser in LAO and HisJ at the equivalent position of Gly97 in NocT which creates a steric hindrance for the accommodation of nopaline and pyronopaline might prevent these molecules from competing with arginine, ornithine or histidine for being bound by their respective PBPs.

The NocT structure is proposed as a reference for further structural comparisons with PBPs involved in the sensing and uptake of other opines, such as agrocinopine [15] and octopine [35]. The opine octopine is a condensate of arginine and pyruvate, therefore structurally close to nopaline. In *A. tumefaciens* strains harbouring an octopine-type Ti plasmid, octopine is recognized by the PBP OccJ and then transported into the cytoplasm where it acts both as a nutrient and a conjugative signal [25]. Interestingly, NocT is involved in the uptake of nopaline and that of octopine in the presence of nopaline; in contrast, OccJ is involved in the uptake of octopine but not nopaline [25], [26]. Although OccJ and NocT share little homology (48% of identity) 8 of the 12 residues interacting with nopaline in NocT are conserved in OccJ. Based on the 4 differences we defined a nopaline-binding signature and highlighted the importance of Gly97 in NocT for spatial accommodation of nopaline/pyronopaline. This signature notably explains why among LAO, HisJ, NocT and OccJ - which all share strong similarities for the arginine moiety of their ligand- NocT is unique in its ability to bind nopaline/pyronopaline. Further elucidation of the octopine-liganded structures of the PBPs OccJ and NocT would be informative in order to understand the binding properties of octopine and compare it with those of nopaline. Moreover, as enzymes encoded by the *noc* genes are able, once activated, to catabolize octopine [25], [26], it would be of great interest to investigate the mechanisms which drive the specific assimilation of nopaline and octopine in *A. tumefaciens* C58, and notably the role of the transcriptional regulator NocR which controls the expression of the *noc* genes. The question about how co-existing *A. tumefaciens* populations evolved a specialized or generalist capacity in the synthesis and degradation pathways of opines remains to be explored.

Some opines, i.e. nopaline, succinanopine and leucinopine can be spontaneously converted into cyclic derivatives under acidic conditions [4], [16], [17]. Pyronopaline was detected in tumours induced by *A. tumefaciens* C58 on the monocot *Asparagus officinalis* [17], but its occurrence was not explored further in other plant hosts. In this work, pyronopaline was not detected in the tomato plant-tumours induced by the same pathogen (i.e. *A. tumefaciens* strain C58), suggesting that plant genotype and/or growth conditions may influence the nopaline/pyronopaline conversion *in vivo*. Even though the formation of the gamma-lactam ring strongly modifies the α-KG part of nopaline, we showed that NocT bound pyronopaline with an affinity higher than that it exhibited for nopaline. Interestingly, nopaline formed with NocT one more polar protein interactions than pyronopaline. However, these interactions were shorter for the pyronopaline. In terms of affinity, the present study highlighted the important contribution of hydrophobic contacts as the positive enthalpy of 2.1 kcal/mol indicated. The tight contacts of the Met117 side chain with the pyronopaline probably made this ligand conformation preferential for NocT. A combination of transcriptomics and genetics established that *A. tumefaciens* C58 assimilated nopaline and pyronopaline *via* the same Ti-plasmid encoded pathway. Hence, regardless of the nopaline/pyronopaline equilibrium in the plant tumours, *A. tumefaciens* appears to mobilize a unique binding, transport, and assimilation system to use them as nutrients.

With respect to the T-DNA/opine-mediated niche construction process, this work experimentally evidenced the validity of the opine concept [3], [4] within the natural agrobacterial environment that the plant tumour is. Indeed, we demonstrated that binding and assimilation of the opine nopaline contributed to the fitness of *A. tumefaciens* strain C58 in the plant tumour when nopaline assimilating and non-assimilating bacteria were co-infected. On the opposite, when both bacterial types were infected separately, they multiplied to reach a similar level in the plant tumour. The plant tumour is a nutrient-rich environment in which sugars, amino acids, phosphate and sulfate accumulate [40]. This observation supports the notion that the presence of nopaline, made available for bacteria in tumours, does not increase the carrying capacity of plant-tumour habitat for *Agrobacterium* pathogens, but selects those able to assimilate it. Using transgenic plants producing opines in all plant tissues but free of *Agrobacterium*-induced tumour, several studies investigated colonization and fitness of opine assimilating and non-assimilating *Pseudomonas* in rhizosphere and phyllosphere [10], [12], [13]. In all the opine-rich compartments, opine assimilating *Pseudomonas* outcompeted non-assimilating *Pseudomonas*. In the same reports, the carrying capacity of the plant tissues was only increased in the carbon-poor phyllosphere compartment, but was not affected in the carbon-rich rhizosphere compartment. The magnitude of the nutrient bias induced by opines seems therefore to directly influence the dynamics and fitness of the bacterial populations. However, it was previously reported that opine accumulation in plant tumour may favor its translocation to other parts of the host plant [41]. This suggests that opines which are stocked in plant tissues could benefit to the pathogen at different times of the infection cycle even after the death of the host plant, by allowing the maintenance of virulent populations (carrying a Ti-plasmid) in soil until the infection of a new host.

## Materials and Methods

### Purification of NocT and its constructed mutants

Using 5′-GGAATTCCATATGAAGGACTACAAAAGCATT and 5′-TTTGCGGCCGCTTAATGGTGATGGTGATGGTGCTGCTTGGGGGAGGCGTC primers, *nocT* gene of *A. tumefaciens* C58 was amplified and then cloned into pET-9aSN1 expression vector (a gift from S. Chéruel, IBBMC, University Paris Sud, Orsay, France). The pET-9aSN1-nocT was used as template to generate directed mutations with QuikChange II XL directed mutagenesis kit (Stratagene). For mutations Met117 to Ser (M117S) and Asn (M117N), the synthetic forward primers 5′–TATCTCCTCAGCCCGAGTACGTTCTTG and 5′-TATCTCCTCACGCCGAATACGTTCTTG and their reverse complemented primers were designed. The nucleotide sequence of all alleles was confirmed by DNA-sequence analysis (GATC, France).

*E. coli* Rosetta pLysS (Merck) was transformed by the recombinant plasmid. Cells were grown at 37°C in tryptone-yeast extract (TY) broth supplemented with 0.5 mM IPTG to induce NocT production, then centrifuged, resuspended in a buffer that consisted in 50 mM Tris–HCl, pH 8.0, 20 mM imidazole and 500 mM NaCl and disrupted by sonication. After centrifugation at 20 000 g for 30 min at 4°C, the supernatant was loaded onto a 5 mL Ni-NTA agarose column (GE Healthcare). Elution of NocT was performed with the following buffer: 50 mM Tris-HCl pH 8.0, 300 mM imidazole and 500 mM NaCl. NocT containing fractions were loaded onto a gel filtration column (HiLoad 26/60 Superdex 200 prep grade, GE Healthcare) equilibrated with 50 mM Tris-HCl pH 8.0 and 150 mM NaCl. NocT (29 221.6 Da including His tag without the signal peptide) was concentrated to 80 mg/mL (2.73 mM).

### Purification, synthesis and quantification of opines

Nopaline was extracted from crushed tomato plant tumour tissues and purified according to the procedure described by Tempé [42]. Pyronopaline was obtained by synthesis using arginine and α-KG as precursors in the presence of sodium cyanoborohydride as described by Tempé [42]. This synthesis resulted in a mix of nopaline and pyronopaline. The complete conversion of this mix into pyronopaline was obtained as described previously [17]. The quantification of nopaline was performed as previously described [31] from macerates of whole tomato tumours.

All solutions of nopaline, pyronopaline and nopaline/pyronopaline were checked by mass spectrometry. The mass spectrometry measurements were performed in negative mode with an electrospray Q/TOF mass spectrometer (Q/TOF Premier, Waters) equipped with the Nanomate device (Advion) with compounds diluted in 50% acetonitrile and 1% formic acid. The Mass Lynx 4.1 software was used for acquisition and data processing. The external calibration was performed with NaI clusters (2 µg/µL, isopropanol/H2O 50/50, Waters) in the acquisition m/z mass range and the estimated mass accuracy is ±0.01 Da (at 300 Da). The spectra of nopaline and pyronopaline are shown in the figure S5.

### Crystallization and structure determination

Crystallization conditions for unliganded (40 mg/mL or 1.37 mM) and liganded NocT (solution of 2.73 mM protein and 8 mM ligand) as well as for the M117N mutant were screened in sitting-drop vapour-diffusion experiments using the Classics and PEG II kits from Qiagen on a nanodrop robot (Cartesian, Proteomic Solution). Condition 34 from Classics was manually optimized at 293 K with home made solution in hanging drops composed of a 1∶1 volume ratio of crystallization solution (2 M Ammonium Sulfate (AS), 0.1 M Na Citrate pH 5.6, 0.2 M K tartrate and 5% PEG 400). Similar manually optimization of condition 31 from PEG II (30% PEG 4000, 0.1 M Tris pH 8 and 0.1M LiSO_4_) for both WT and mutant liganded proteins led to plate-shaped crystals. Crystals were transferred to a cryo-protectant solution (paraffin oil for AS precipitant or 20% (w/v) PEG 400 for PEG precipitant) and flash-frozen in liquid nitrogen. Diffraction data were collected at 100 K on the PROXIMA I beamline at SOLEIL synchrotron (Saint-Aubin, France). Data collection and processing statistics are given in Table S1. Structure determination of all crystals were performed by molecular replacement with PHASER [43] using first the coordinates of the N-terminal (residues 1–86 and 197–238) and the C-terminal (residues 93–185) of the *E.coli* histidine binding protein HisJ (1HSL) as two search models for the free-liganded structure and next our NocT model for the wild-type or mutant liganded-NocT complexes. Refinement was performed with BUSTER-2.10 [44] with NCS restraints as all asymmetric units contain two protein molecules. One TLS group was assigned for each structure. Electron density maps were evaluated using COOT [45]. Refinement details are shown in Table S1. Molecular graphics images were generated using PYMOL (http://www.pymol.org).

### *K_D_* measurements by fluorescence titration and microcalorimetry

Ligand-NocT interaction was monitored by autofluorescence by exciting the protein at a wavelength of 295 nm and monitoring the quenching of fluorescence emission of tryptophan residues at 340 nm. All experiments were performed at 25°C in 25 mM Tris-HCl pH 8.0 and 150 mM NaCl with a fixed amount of proteins (5 µM) and increasing concentrations of ligand using a SpectraMax M5 microplate reader (Molecular Devices). Each ligand exhibited no emission signal at 340 nm. Fluorescence measurements were done in triplicates. The data were analysed using Origin 7 software and fitted to the equation f = ΔFluorescence_max_ * abs(x)/(KD+abs(x)).

ITC experiments were performed with an ITC200 isothermal titration calorimeter from MicroCal Llc (Northampton, MA). The experiments were carried out at 20°C. Protein concentration in the microcalorimeter cell (0.2 ml) varied from 100 to 150 µM. Nineteen injections of 2 µl of the ligand solution concentration from 1.5 to 1.6 mM were performed at intervals of 180 s while stirring at 1000 rpm. The experimental data were fitted to theoretical titration curves with software supplied by MicroCal (ORIGIN). This software uses the relationship between the heat generated by each injection and ΔH (enthalpy change in Kcal.mol^−1^), Ka (the association binding constant in M^−1^), n (the number of binding sites), total protein concentration and free and total ligand concentrations.

### Differential scanning calorimetry

Thermal stability of wild type and M117N-NocT (20.5 µM) was studied by differential scanning calorimetry (DSC) on a MicroCal model VP-DSC in a standard buffer. Each measurement was preceded by a baseline scan with the standard buffer. All solutions were degassed just before loading into the calorimeter. Scans were performed at 1K.min-1 between 20 and 90°C. The heat capacity of the buffer was subtracted from that of the protein sample before analysis. Thermodynamic parameters were determined by fitting the data to the following equation ΔC_p_(T) = (K_d_(T) ΔH_cal_ ΔH_vH_)/((1+K_d_(T))^2^ RT^2^) where K_d_ is the equilibrium constant for a two-state process, ΔH_vH_ is the enthalpy calculated on the basis of a two-state process and ΔH_cal_ is the measured enthalpy.

### Growth conditions and construction of the *A. tumefaciens* C58 mutants

The *A. tumefaciens* C58 derivatives carrying pTi-accR::Gm and pTi::Gm (used as C58-control in plant assay) in which the genes *accR* (*atu6138*) and *atu6147* were disrupted by inserting a gentamicin resistance cassette were already constructed [46]. The *A. tumefaciens* C58 derivatives harbouring the Ti-plasmid pTi-nos::Km, pTi-ocd::Gm, pTi-nocT::Gm were obtained by insertion of a gentamicin (Gm) or kanamycin (Km) resistance cassette into the genes *nos* ( = *atu6015*), *ocd* ( = *atu6016*), and *nocT* ( = *atu6027*) as described previously [46].

*A. tumefaciens* was cultivated at 30°C in *Agrobacterium* broth (AB) minimal medium supplemented with ammonium chloride (1 g/L) and mannitol (2 g/L) except when an alternative source of carbon and nitrogen is indicated, or in Luria-Bertani modified medium (LBm; NaCl 5 g/L). In growth assay nopaline and pyronopaline were added as a sole carbon and nitrogen source at 3 mM (ca 1 g/L). The antibiotics gentamycin and kanamycin were added at 25 µg/mL and 50 µg/mL, respectively.

### Transcriptomic analysis

Overnight cultures of the *A. tumefaciens accR* mutant were sub-cultured at an initial OD_600_ of 0.05 in 50 ml of AB medium containing ammonium chloride (1 g/L) and mannitol (2 g/L) which was supplemented or not with the mix nopaline/pyronopaline at 1 mM. Cells were grown at 28°C for approximately 9 hours until early exponential phase (OD_600_ = 0.4). RNA extraction was performed using a phenol-based procedure, according to Planamente *et al.* [19]. Construction of cDNA libraries, hybridization and signal quantification were performed by the PartnerChip platform (Génopole Evry, France). Experiments were performed in triplicates. Normalized data of samples were pairwise compared and P values corresponding to statistical t-test were attributed for each gene.

### Plant infection and numeration of bacterial populations

Tomato plants (F1 hybrid Dona, Vilmorin, France) were grown in greenhouse under long day conditions and controlled temperature (24–26°C). One-month old plants were scalpel wounded between first and second stem nodes and inoculated with the agrobacteria as described previously [20]. For each independent experiment, five to seven plant tumours (32 dpi) were crushed into NaCl 0.8% to recover the bacteria which were then spotted onto selective agar media to enumerate colony forming units (CFU). In the case of mixed infections, the proportions of the genotypes (C58-control and *nos*, *nocT* and *ocd* KO-mutants) were measured in the inoculum (P_i_) and the plant tumour (P_t_) using antibiotic resistances and PCR with appropriate primers. This allowed calculation of the competitive index CI = (P_t_^mutant^/P_t_^control^)/(P_i_^mutant^/P_i_^control^) as described by Macho et al. [47]. The *A. tumefaciens* derivative C58.00 that contains no plasmid but harbours a chromosomal resistance to rifampicin, was used as a recipient strain. The transconjugants that acquired a Ti-plasmid from the donor strains were enumerated using antibiotic resistances and PCR with appropriate primers.

### Phylogenetic analysis

Sequences were analyzed using blastP from NCBI (http://blast.ncbi.nlm.nih.gov/) and MicroScope (https://www.genoscope.cns.fr/agc/microscope/about/collabprojects.php). Alignements of NocT and related sequences were conducted using the ClustalW software. Relationship tree was build using the MEGA software, Version 5. The phylogeny was inferred using the neighbor-joining method. The bootstrap consensus tree inferred from 1000 replicates was taken to represent the evolutionary history of the taxa analyzed. The evolutionary distances are in units of the number of amino acid substitutions per site.

### Accession codes

The atomic coordinates and structure factors of the unliganded NocT and the complexes with nopaline and pyronopaline as well as the M117N-NocT in complex with pyronopaline have been deposited in the protein data bank (http://www.rcsb.org) under accession codes 4P0I, 4POX, 4POW and 4PP0 respectively.

## Supporting Information

Figure S1 Structural comparison between NocT-nopaline vs NocT-pyronopaline. Structural comparison between the binding sites of NocT in complex with nopaline (shown as pink/limegreen stick) and pyronopaline (shown as pink/blue stick). Close-up view around the α-KG part of both ligands. The region 234–238 and the Met117, His170, Ser169 and Gln99 positions are affected by the type of bound ligand.
(PDF)

Figure S2 ITC and Fluorescence *K_D_* measurements. NocT fluorescence monitoring upon titration with each ligand and fit (solid line) to a single binding model using Origin software. NocT ITC measurements: the top panel shows heat differences upon injection of ligand and lower panel show integrated heats of injection and the best fit (solid line) to a single binding model using Microcal Origin. Measures were done in triplicates.
(PDF)

Figure S3 Differential scanning calorimetry (DSC) thermogram of NocT and NocT-M117N.
(PDF)

Figure S4 Structural comparison between NocT vs NocT-M117N. A, Structural comparison between the binding sites of NocT in complex with nopaline (shown as pink/limegreen stick) and pyronopaline (shown as pink/blue stick) and NocT-M117N mutant in complex with pyronopaline (shown as pink/magenta stick). Close-up view around the α-KG part of the ligand; B, pyronopaline bound to the ligand binding site of M117N-NocT in its simulated annealing Fo-Fc omit map contoured at 4 σ.
(PDF)

Figure S5 ESI-TOF Mass spectrometry of nopaline and pyronopaline. The calculated molecular weight is indicated as MWc.
(PDF)

Table S1 Crystallographic data and refinement parameters.
(PDF)

Table S2 Transcriptomic data in the presence vs absence of nopaline/pyronopaline.
(PDF)
