# Supplementary material for: Agrobacterium Uses a Unique Ligand-Binding Mode for Trapping Opines and Acquiring A Competitive Advantage in the Niche Construction on Plant Host
Source: PLoS Pathog. 2014 Oct 9;10(10):e1004444. doi: 10.1371/journal.ppat.1004444 (PMC4192606; doi:10.1371/journal.ppat.1004444)
Supplement: Figure S1 — Structural comparison between NocT-nopaline vs NocT-pyronopaline. Structural comparison between the binding sites of NocT in complex with nopaline (shown as pink/limegreen stick) and pyronopaline (shown as pink/blue stick). Close-up view around the α-KG part of both ligands. The region 234–238 and the Met117, His170, Ser169 and Gln99 positions are affected by the type of bound ligand. (PDF) [file ppat.1004444.s001.pdf]

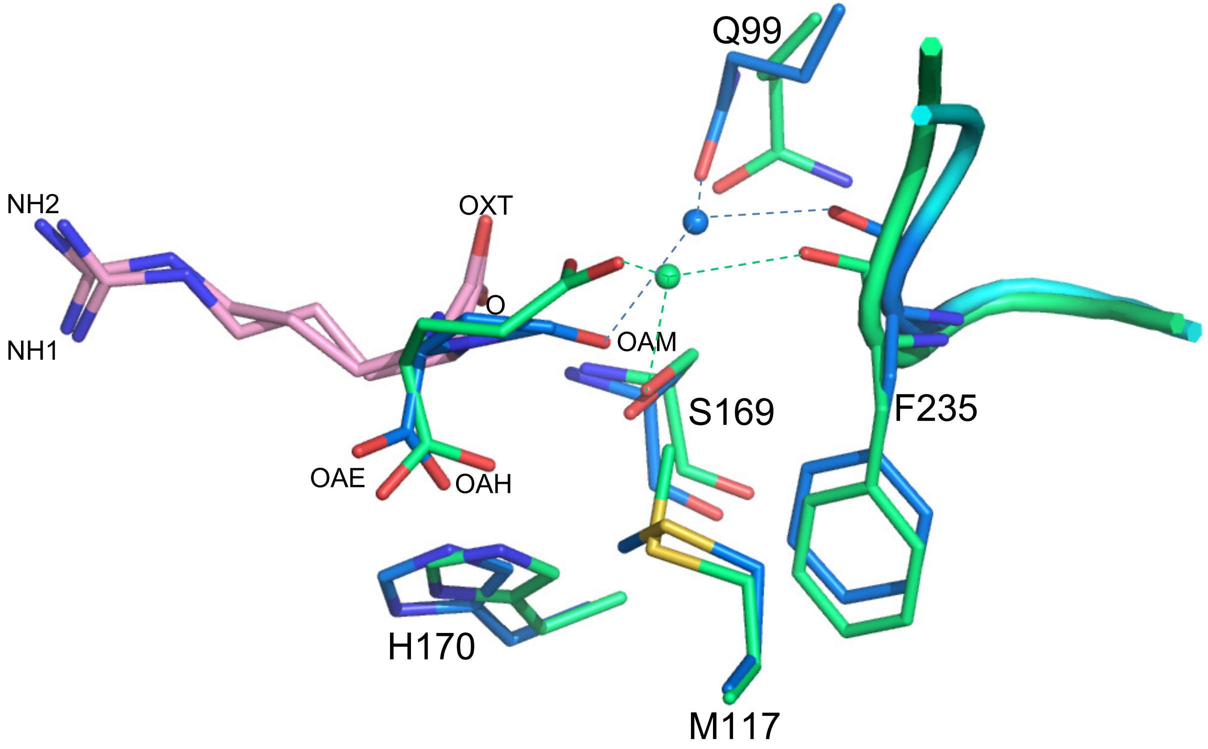

Figure S1. Structural comparison between NocT-nopaline vs NocT-pyronopaline. Structural comparison between the binding sites of NocT in complex with nopaline (shown as pink/limegreen stick) and pyronopaline (shown as pink/blue stick). Close-up view around the α-KG part of both ligands. The region 234-238 and the Met117, His170, Ser169 and Gln99 positions are affected by the type of bound ligand.
