# Supplementary material for: Agrobacterium Uses a Unique Ligand-Binding Mode for Trapping Opines and Acquiring A Competitive Advantage in the Niche Construction on Plant Host
Source: PLoS Pathog. 2014 Oct 9;10(10):e1004444. doi: 10.1371/journal.ppat.1004444 (PMC4192606; doi:10.1371/journal.ppat.1004444)
Supplement: Figure S2 — ITC and Fluorescence KD measurements. NocT fluorescence monitoring upon titration with each ligand and fit (solid line) to a single binding model using Origin software. NocT ITC measurements: the top panel shows heat differences upon injection of ligand and lower panel show integrated heats of injection and the best fit (solid line) to a single binding model using Microcal Origin. Measures were done in triplicates. (PDF) [file ppat.1004444.s002.pdf]

## Fluorescence

| NocT$_{wt}$-Nopaline | NocT$_{wt}$-Pyronopaline | NocT$_{M117S}$-Pyronopaline | NocT$_{M117N}$-Pyronopaline | NocT$_{wt}$-Arginine |
|---|---|---|---|---|
| $K_D$ =3.7 ± 0.6 µM | $K_D$ = 0.5 ± 0.07 µM | $K_D$ = 37.8 ± 9 µM | $K_D$ = 39.9 ± 7 µM | No interaction |
| 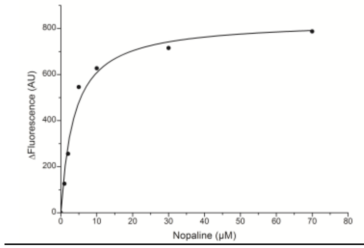 | 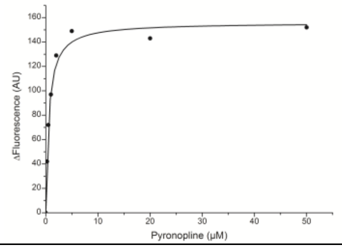 | 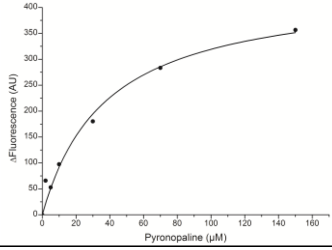 | 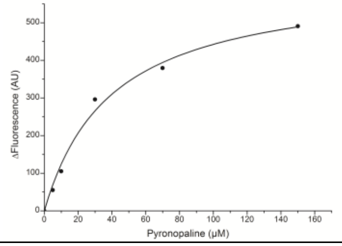 | 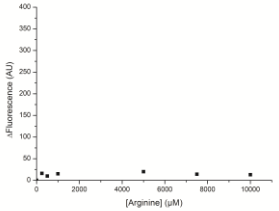 |

## ITC

| NocT$_{wt}$-Nopaline | NocT$_{wt}$-Pyronopaline | NocT$_{wt}$-Arginine | NoctT$_{wt}$-Histidine | NocT$_{wt}$-Ornithine |
|---|---|---|---|---|
| | $K_D$ = 0.58 ± 0.05 µM | No interaction | No interaction | No interaction |
| Non Acquired | 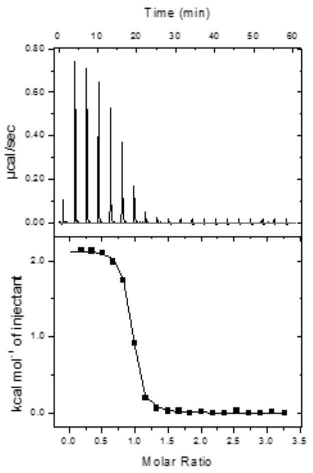 | 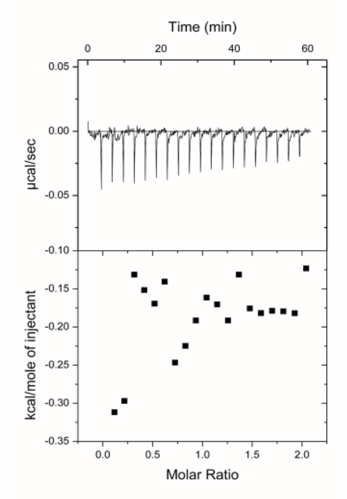 | 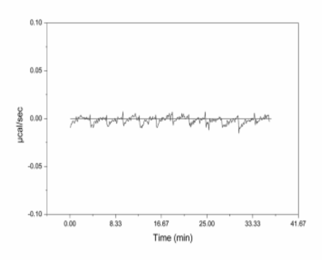 | 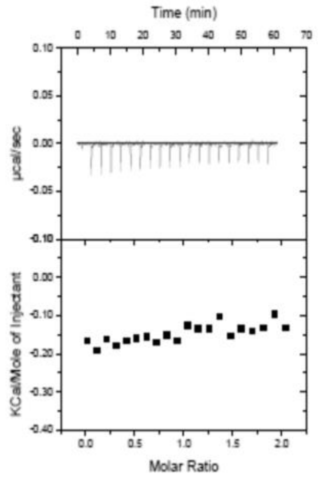 |

ITC and Fluorescence $K_D$ measurements.

NocT fluorescence monitoring upon titration with each ligand and fit (solid line) to a single binding model using Origin. Measures were done in triplicates.

NocT ITC : The top panel show heat differences upon injection of ligand and lower panel show integrated heats of injection and the best fit (solid line) to a single binding model using Microcal Origin.
