# Supplementary figures and images for: Agrobacterium Uses a Unique Ligand-Binding Mode for Trapping Opines and Acquiring A Competitive Advantage in the Niche Construction on Plant Host
Source: PLoS Pathog. 2014 Oct 9;10(10):e1004444. doi: 10.1371/journal.ppat.1004444 (PMC4192606; doi:10.1371/journal.ppat.1004444)

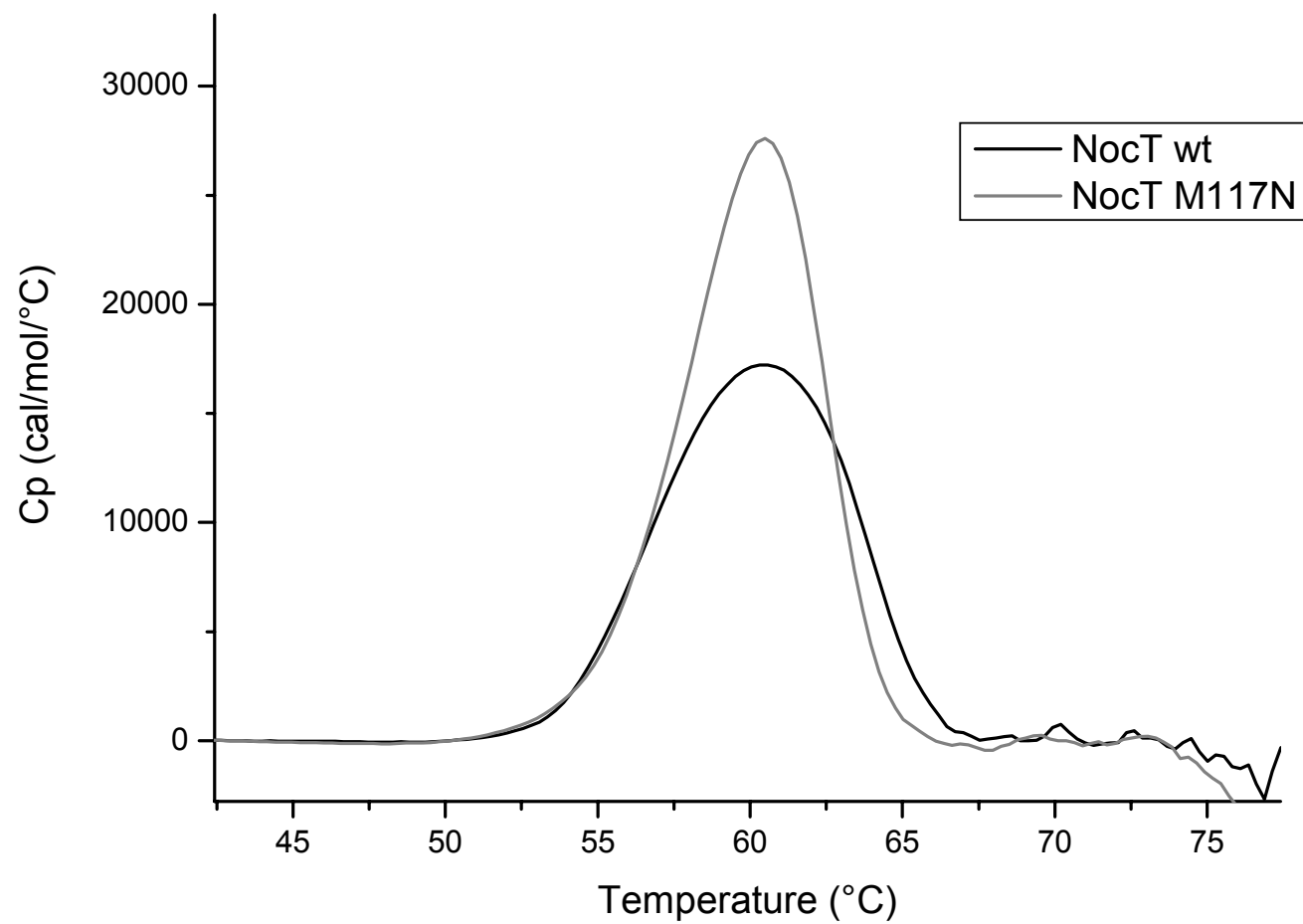

**Figure S3. Differential scanning calorimetry (DSC) thermogram of NocT and NocT-M117N**

Supplement: Figure S3 — Differential scanning calorimetry (DSC) thermogram of NocT and NocT-M117N. (PDF) [file ppat.1004444.s003.pdf]

**Figure S5 Mass spectrometry (M-H) of nopaline and pyronopaline**

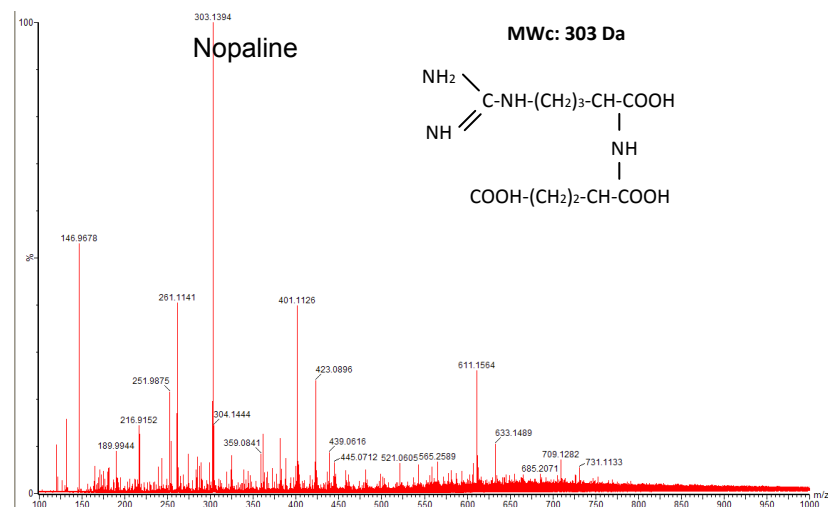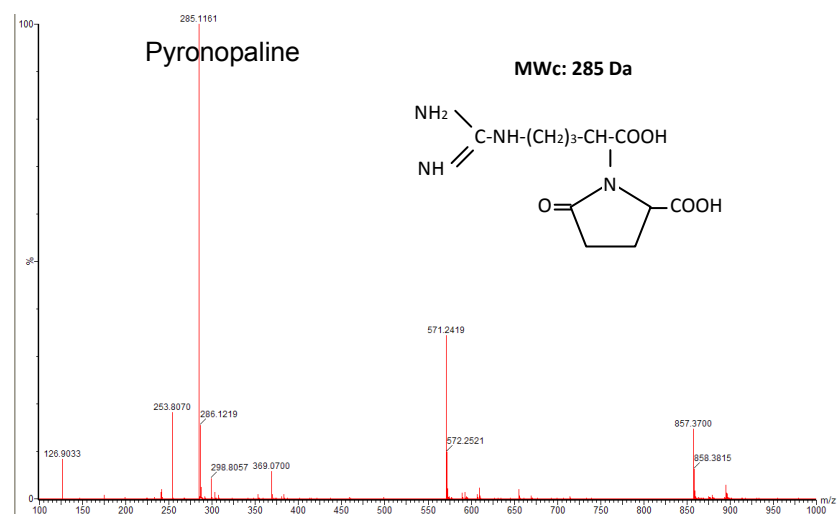

Supplement: Figure S5 — ESI-TOF Mass spectrometry of nopaline and pyronopaline. The calculated molecular weight is indicated as MWc. (PDF) [file ppat.1004444.s005.pdf]
