# Supplementary material for: Agrobacterium Uses a Unique Ligand-Binding Mode for Trapping Opines and Acquiring A Competitive Advantage in the Niche Construction on Plant Host
Source: PLoS Pathog. 2014 Oct 9;10(10):e1004444. doi: 10.1371/journal.ppat.1004444 (PMC4192606; doi:10.1371/journal.ppat.1004444)
Supplement: Figure S4 — Structural comparison between NocT vs NocT-M117N. A, Structural comparison between the binding sites of NocT in complex with nopaline (shown as pink/limegreen stick) and pyronopaline (shown as pink/blue stick) and NocT-M117N mutant in complex with pyronopaline (shown as pink/magenta stick). Close-up view around the α-KG part of the ligand; B, pyronopaline bound to the ligand binding site of M117N-NocT in its simulated annealing Fo-Fc omit map contoured at 4 σ. (PDF) [file ppat.1004444.s004.pdf]

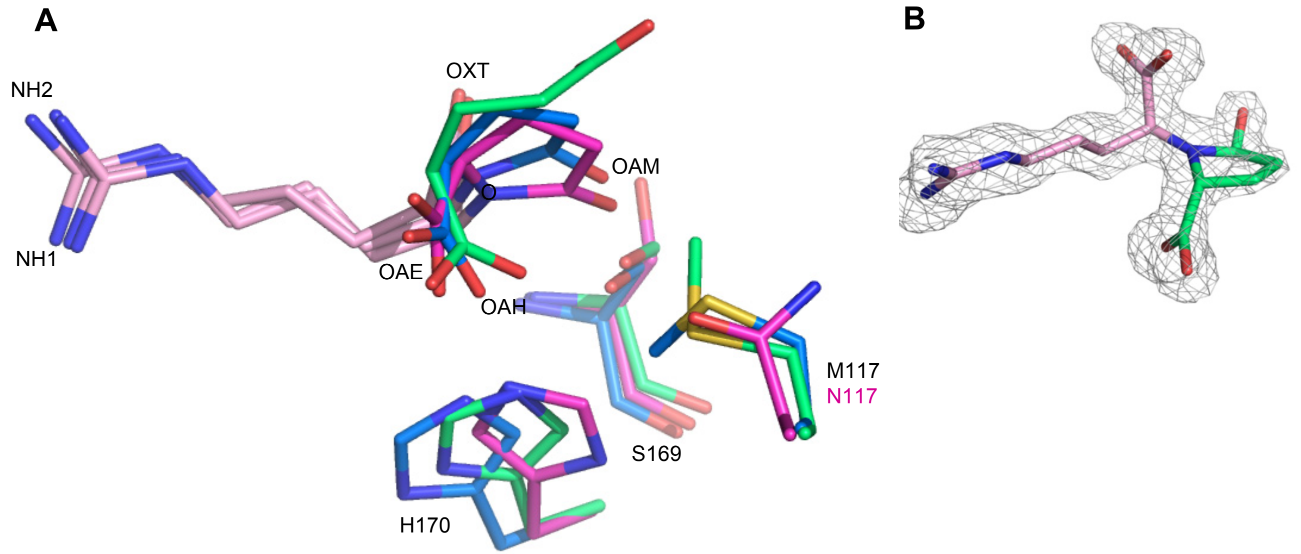

Figure S4. Structural comparison between NocT vs NocT-M117N. A, Structural comparison between the binding sites of NocT in complex with nopaline (shown as pink/limegreen stick) and pyronopaline (shown as pink/blue stick) and M117N-NocT mutant in complex with pyronopaline (shown as pink/magenta stick). Close-up view around the α-KG part of the ligand; **B**, pyronopaline bound to the ligand binding site of M117N-NocT in its annealing Fo-Fc omit map contoured at 4 σ.
