# Supplementary material for: Agrobacterium Uses a Unique Ligand-Binding Mode for Trapping Opines and Acquiring A Competitive Advantage in the Niche Construction on Plant Host
Source: PLoS Pathog. 2014 Oct 9;10(10):e1004444. doi: 10.1371/journal.ppat.1004444 (PMC4192606; doi:10.1371/journal.ppat.1004444)
Supplement: Table S1 — Crystallographic data and refinement parameters. (PDF) [file ppat.1004444.s006.pdf]

Table S1. Crystallographic data and refinement parameters

NocT	Unliganded	Nopaline	Pyronopaline	M117N-Pyronopaline
PDB code	4P0I	4POX	4POW	4PP0
Space group<br>Cell parameters (Å,°)	C2<br>*a* = 118.9<br>*b* = 69.7<br>*c* = 74.4<br>β = 117.9	$P3_2$<br>*a* = 114.4<br>*b* = 114.4<br>*c* = 37.8	$P3_2$<br>*a* = 114.5<br>*b* = 114.5<br>*c* = 37.8	$P3_2$<br>*a* = 114. 6<br>*b* = 114.6<br>*c* = 37.9
Resolution (Å)	38.8- 1.89 (2-1.89)	50- 2.29 (2.43-2.29)	22.7 - 1.55 (1.64- 1.55)	23- 1.57 (1.66-1.57)
No. of observed reflections	259569 (41688)	85996 (13013)	446821 (66404)	437965 (66081)
No. of unique reflections	43103 (6909)	24543 (3717)	80447 (12944)	77698 (12464)
R_{sym} (%)	5.3 (53.4)	10.6 (96.1)	5 (73.6)	4.9 (75)
Completeness (%)	99.4 (99.2)	98.8 (93.1)	99.9 (99.3)	99.8 (99)
I/σ	22.43 (3.61)	9.2 (1.29)	17.3 (2.02)	18.94 (2)
R_{cryst} (%)	20.5	18.9	17	16.7
R_{free} (%)	23.6	21.8	19	18.3
rms bond deviation (Å)	0.01	0.009	0.01	0.01
rms angle deviation (°)	1.06	1.07	1.05	1.03
Average B (Å^2)				
protein	39.4	49.2	29.6	30.8
ligand	-	44.4	23	22
solvent	38.3	47.7	39.6	39.5

Values for the highest resolution shell are in parentheses
