# Supplementary material for: Agrobacterium Uses a Unique Ligand-Binding Mode for Trapping Opines and Acquiring A Competitive Advantage in the Niche Construction on Plant Host
Source: PLoS Pathog. 2014 Oct 9;10(10):e1004444. doi: 10.1371/journal.ppat.1004444 (PMC4192606; doi:10.1371/journal.ppat.1004444)
Supplement: Table S2 — Transcriptomic data in the presence vs absence of nopaline/pyronopaline. (PDF) [file ppat.1004444.s007.pdf]

Table S2. Transcriptomic fold change (Fch>20 in bold face) in *A. tumefaciens accR* mutant in the presence vs absence of nopaline/pyronopaline

atu code	Gene name	FCh µarrays	FCh RT-qPCR	Function / putative function
atu1467		-12.49	-	hypothetical protein
atu1468		-45.97	-	conserved hypothetical protein
atu1643		10.65	-	conserved hypothetical protein
atu1847		6.55	-	hypothetical protein
atu1996		-5.91	-	hypothetical protein
atu1997	*cysJ*	-38.93	-71.94	sulfite reductase
atu2019	*ilvC*	-3.35	-	ketol-acid reductoisomerase
atu2022	*adh*	-3.62	-	NADP-dependent alcohol dehydrogenase
atu2107	*tnp*	6.40	-	IS3 family transposase
atu2224	*aldA*	-4.47	-	aldehyde dehydrogenase
atu4007	***arcA***	**49.90**	98.02	arginase
atu4155		3.46	-	ABC transporter, membrane spanning protein
atu4676		3.53	-	putative malate dehydrogenase
atu4678		3.27	-	ABC transporter, substrate binding protein
atu4732		3.16	-	fimbrial chaperone
atu4858	*traG*	3.53	-	Agrobacterium virulence virD4-like protein
atu6016	***ocd***	**40.42**	-	ornithine cyclodeaminase
atu6017	***odh***	**21.63**	-	NAD/NADP octopine/nopaline dehydrogenase
atu6018	***arc***	**38.32**	-	arginase
atu6019	***noxA***	**20.40**	-	D-nopaline dehydrogenase
atu6020		**45.97**	-	conserved hypothetical protein
atu6021	***noxB***	**35.49**	147.27	D-nopaline dehydrogenase
atu6022	***hyuA***	**46.54**	-	N-methylhydantoinase A/acetone carboxylase, beta subunit
atu6023	***hyuB***	**25.89**	-	N-methylhydantoinase B/acetone carboxylase, alpha subunit
atu6024		5.68	-	transcriptional regulator, LysR family
atu6025	***nocM***	**21.01**	-	ABC transporter, membrane spanning protein [nopaline]
atu6026	***nocQ***	**24.40**	-	ABC transporter, membrane spanning protein [nopaline]
atu6027	***nocT***	**54.47**	68.14	ABC transporter, substrate binding protein [nopaline]
atu6028	***nocP***	**55.03**	99.80	ABC transporter, nucleotide binding/ATPase protein [nopaline]
atu6096		3.15	-	conserved hypothetical protein
atu6097	*fic*	3.29	-	cell filamentation protein
atu8021		-3.53	-	conserved hypothetical protein
